# Deciphering the Contribution of Biofilm to the Pathogenesis of Peritoneal Dialysis Infections: Characterization and Microbial Behaviour on Dialysis Fluids

**DOI:** 10.1371/journal.pone.0157870

**Published:** 2016-06-23

**Authors:** Joana Sampaio, Diana Machado, Ana Marta Gomes, Idalina Machado, Cledir Santos, Nelson Lima, Maria João Carvalho, António Cabrita, Anabela Rodrigues, Margarida Martins

**Affiliations:** 1 CEB – Centre of Biological Engineering, LIBRO – Laboratório de Investigação em Biofilmes Rosário Oliveira, University of Minho, Campus de Gualtar, Braga, Portugal; 2 Serviço Nefrologia, Centro Hospitalar Vila Nova de Gaia/Espinho, Rua Conceição Fernandes, Vila Nova de Gaia, Portugal; 3 LEPABE, Department of Chemical Engineering, Faculty of Engineering, University of Porto, Porto, Portugal; 4 Department of Chemical Sciences and Natural Resources, CIBAMA, BIOREN-UFRO Universidad de La Frontera, Temuco, Chile; 5 CEB – Centre of Biological Engineering, Micoteca da Universidade do Minho, Campus de Gualtar, Braga, Portugal; 6 Serviço Nefrologia, Centro Hospitalar Porto, Largo Prof. Abel Salazar Porto, Porto, Portugal; 7 Unidade Multidisciplinar de Investigação Biomédica, Instituto de Ciências Biomédicas Abel Salazar, Universidade do Porto - UMIB/ICBAS/UP, Porto, Portugal; Laurentian, CANADA

## Abstract

Infections are major complications in peritoneal dialysis (PD) with a multifactorial etiology that comprises patient, microbial and dialytic factors. This study aimed at investigating the contribution of microbial biofilms on PD catheters to recalcitrant infections and their interplay with PD related-factors. A prospective observational study was performed on 47 patients attending Centro Hospitalar of Porto and Vila Nova de Gaia/Espinho to whom the catheter was removed due to infectious (n = 16) and non-infectious causes (n = 31). Microbial density on the catheter was assessed by culture methods and the isolated microorganisms identified by matrix-assisted laser desorption/ionization time-of-flight intact cell mass spectrometry. The effect of conventional and three biocompatible PD solutions on 16 Coagulase Negative Staphylococci (CNS) and 10 *Pseudomonas aeruginosa* strains planktonic growth and biofilm formation was evaluated. Cultures were positive in 87.5% of the catheters removed due infectious and 90.3% removed due to non-infectious causes. However, microbial yields were higher on the cuffs of catheters removed due to infection vs. non-infection. Staphylococci (CNS and *Staphylococcus aureus*) and *P*. *aeruginosa* were the predominant species: 32% and 20% in the infection and 43.3% and 22.7% in the non-infection group, respectively. In general, PD solutions had a detrimental effect on planktonic CNS and *P*. *aeruginosa* strains growth. All strains formed biofilms in the presence of PD solutions. The solutions had a more detrimental effect on *P*. *aeruginosa* than CNS strains. No major differences were observed between conventional and biocompatible solutions, although in icodextrin solution biofilm biomass was lower than in bicarbonate/lactate solution. Overall, we show that microbial biofilm is universal in PD catheters with the subclinical menace of Staphylococci and *P*. *aeruginosa*. Cuffs colonization may significantly contribute to infection. PD solutions differentially impact microbial species. This knowledge is important for the development of infection diagnosis, treatment and preventive strategies.

## Introduction

Peritoneal dialysis (PD) is a beneficial therapeutic option for end-stage renal disease patients, with favourable survival outcomes in comparison with hemodialysis [1]. However, infections are major complications of PD. Catheter (exit-site/ tunnel) infections, ranging between 0.11–0.93 episodes/patient-year [2], are associated with high risk of developing catheter-related peritonitis [3]. Therefore, 15–57% of these episodes result in catheter removal and technique failure [4]. Peritonitis, ranging between 0.06–1.66 episodes/patient-year, is responsible for ~20% of the cases of PD discontinuation, also contributing to residual renal function loss, long-term peritoneal membrane injury and 2–6% of deaths [5]. Although until a few years ago the incidence of PD infections decreased significantly [6] due to technique improvements and patients care, nowadays only modest improvements are observed in cure and catheter salvage [7]. Indeed, 20–30% of the catheter-related infections are not cured [8], leading to chronic infections.

The pathogenesis of catheter-related infections depends on the interplay between patient, microbial and dialytic factors.

First, the population factors associated with these infections are not clear, although gender, time on PD [9], age [10] or uraemia [11] have been highlighted as potential risk factors.

Second, chronic infections have been associated with specific microorganisms. Relapsing peritonitis (same microorganism or culture-negative episode within 4 weeks of completion of therapy for a prior episode) has been associated with *Pseudomonas* [10] or Staphylococci (Coagulase-Negative Staphylococci, CNS, and *Staphylococcus aureus*) [11]. Recurrent peritonitis (different microorganism within 4 weeks of completion of therapy for a prior episode) has been associated with *Enterococcus* species, Gram-negative microorganisms, polymicrobial cultures [10] or fungi [11]. Repeat peritonitis (same microorganism occurring more than 4 weeks after completion of therapy for a prior episode) has been associated with Staphylococci [12, 13]. It has been suggested that microorganisms can develop antimicrobial resistance during treatment course or exhibit distinct virulence factors, as biofilm formation ability. Biofilms are communities of microorganisms adhered to a surface and enmeshed in an extracellular matrix of microbial and host-derived components. In contrast to cells in suspension (i.e., planktonic), this protective phenotype enables microorganisms to evade the immune system and to gain tolerance to antimicrobial drugs. Furthermore, microorganisms on biofilms can be sloughed, contributing to the persistence of the infection [14]. Biofilms in PD catheters have been demonstrated by microscopy and culture methods [15] and indirectly by the presence of DNA fragments on the dialysis fluid [16]. It should be noted that although the presence of biofilms in PD catheters is generally accepted, its clinical significance is not understood [17].

Third, the impact of dialytic factors on these recalcitrant infections is poorly investigated. However, automatic PD (APD) has been associated with a reduced risk of developing a first Gram-positive peritonitis but with higher risk of Gram-negative peritonitis vs. continuous ambulatory PD (CAPD) [18]. On the other hand, PD solutions are critical for solute and electrolyte exchanges. Lately, conventional solutions (acidic, with lactate, high glucose concentration and high level of glucose breakdown products) have started to be replaced by biocompatible solutions with a neutral pH, bicarbonate or lactate/bicarbonate and low glucose or icodextrin, presenting low/medium glucose breakdown products [19]. Current evidences do not conclusively support benefits of the use of biocompatible solutions on peritonitis outcomes [20]. Both types of solutions have been suggested to inhibit planktonic microorganisms growth. Conventional solutions do not impair biofilm formation [15], but it is not known how biocompatible solutions affect this parameter.

Due to the relevance of PD catheter-related infections and the remarkable lack of understanding of the contribution of biofilms to their pathogenesis, this laboratory study evaluated (i) the microbial density and etiology of biofilms on PD catheters in patients with and without infection, (ii) infection-associated patient factors and (iii) the impact of PD solutions on microorganisms’ survival.

## Material and Methods

### Population

**Fig 1:**
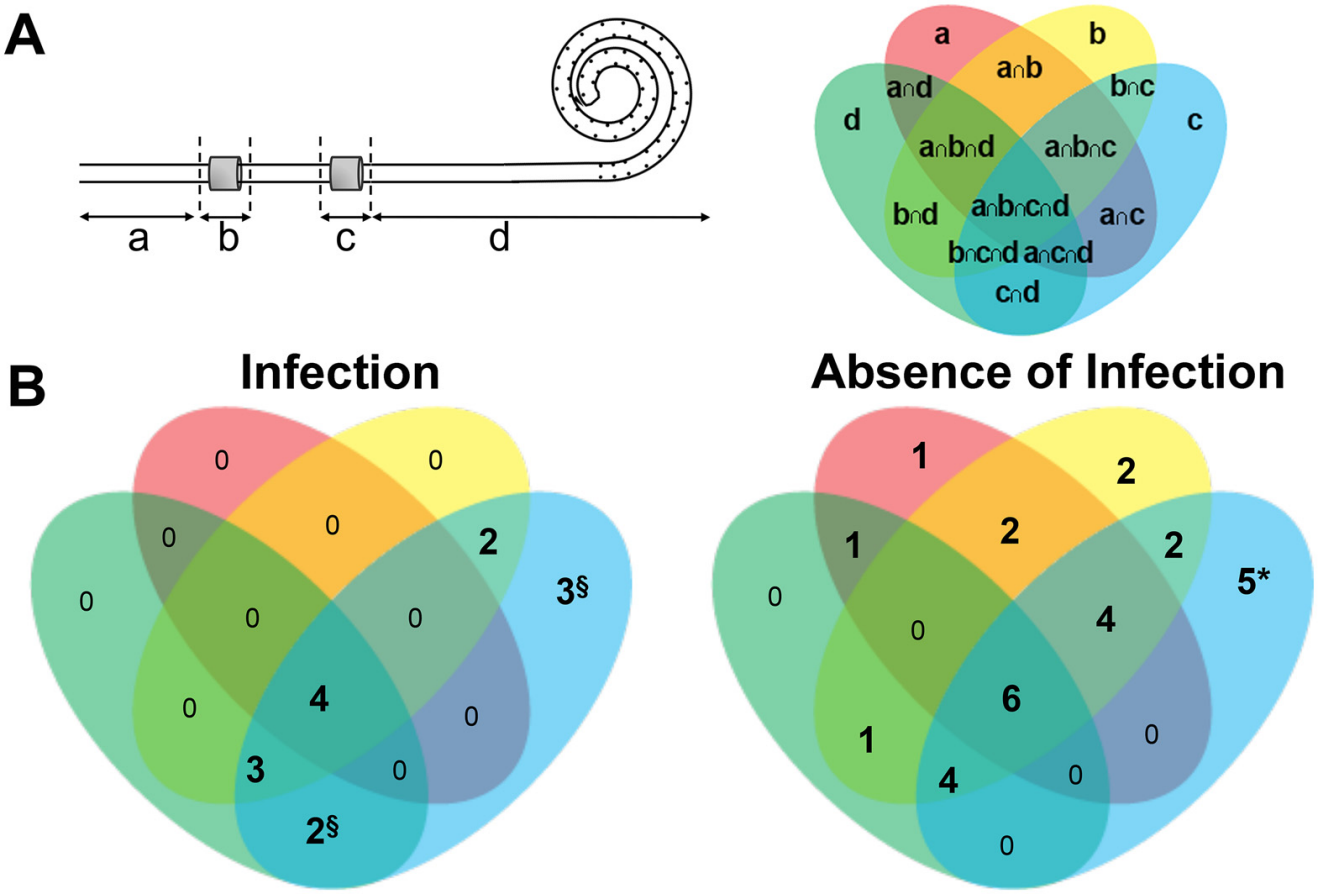
PD catheters. Schematic diagram of the catheter segments including the silicone based segments: external **(a)** and intraperitoneal **(d)** and the cuffs: subcutaneous **(b)** and deep **(c)** (left). The different segments were cultured separately. Therefore, a Venn diagram (right) illustrates the possible relationships among them (external segment in red, subcutaneous cuff in yellow, deep cuff in blue and intraperitoneal segment in green) **(A)**. The representation of the distribution and overlap of positive segment cultures in catheters removed from patients with (left) and without infection (right) **(B)**. Values represent the number of catheters with a specific catheter colonization pattern. Four patients had growth on all catheter segments—with refractory and fungal (n = 3) and catheter-related peritonitis (n = 1); 2 patients had growth on the catheter subcutaneous and deep cuffs—with relapsing peritonitis (n = 1) and chronic catheter infection (n = 1); 3 patients had growth only on the catheter deep cuff—with relapsing peritonitis (n = 1) and chronic catheter infection (n = 2); 2 patients had growth on the catheter deep cuff and intraperitoneal segment—with chronic catheter infection; 3 patients had growth on the catheter subcutaneous and deep cuffs and intraperitoneal segment—with catheter-related peritonitis (n = 1), chronic catheter infection (n = 2). At the time of removal, ^§^2 and *, 1 catheter did not present subcutaneous cuff.

This study was approved by the Ethics Committee of Centro Hospitalar do Porto [243/11(153-DEFI/229-CES)] and was performed in accordance with Declaration of Helsinki [21]. The International Society for Peritoneal Dialysis infection criteria were applied [17]. Between September 2011 and June 2013, we included 47 patients on PD at the Division of Nephrology of Centro Hospitalar do Porto-Hospital Santo Antonio and Vila Nova Gaia/Espinho whose catheter was removed by clinical decision (S1 Table). Patients provided written informed consent. Population characteristics and microorganisms recovered from the exit-site/tunnel and/or peritoneal fluid were obtained from medical records. All patients with infection received appropriated treatment prior to catheter removal. All catheters were removed in a surgical environment, divided into external, intraperitoneal segments, subcutaneous and deep cuff (Fig 1A), placed separately in sterile bags and transported at 4°C for microbiological processing on the same day.

### Microbial density and species on catheter segments

The isolation protocol was designed in order to recover the microorganisms most frequently associated with infections. External segments outer surface was disinfected with ethanol as previously described [22]. The segment was split longitudinally and the biomass on the inner surface recovered by scrapping and resuspended in 400 μL of Tryptic Soy Broth (TSB, Liofilchem). Intraperitoneal segments were split longitudinally, cut in pieces and placed in TSB (4 mL). Cuffs were placed in TSB (4 mL). All the suspensions were sonicated at 50–60 Hz (Sonicor) during 5 min and vortexed for 30 s. For semi-quantitative analysis, 100 μL of the suspension was plated onto Tryptose Blood Agar Base (Oxoid)-7% defibrinated sheep blood (Probiologica) (TBA) and incubated at 37°C, 72 h. The remaining suspension was inoculated in additional 5 mL of TSB—7% defibrinated sheep blood and incubated at 37°C, 120 rpm for 24 h, followed by subculturing on TBA at 37°C, up to 72 h. Two independent researchers recorded the number of colony forming units (CFU), examined the colonies features (for elevation and margin) and picked up several colonies for further identification. The detection limits were of 4 CFU/ external segment and of 40 CFU/ intraperitoneal segment or cuff.

All the isolated microorganisms were identified by matrix-assisted laser desorption/ionization time-of-flight intact cell mass spectrometry (MALDI-TOF-ICMS). Briefly, all the strains were grown in TBA at 37°C for 24 h. The sample was prepared by the direct transfer procedure, consisting on the smear of one colony in a well of the target plate and mixing it with 0.5 μL of the MALDI matrix solution [75 mg/ml 2,5-dihydroxybenzoic acid (Sigma) in ethanol (Sigma) /water/acetonitrile (Sigma) [1:1:1] with 0.03% trifluoroacetic acid (Sigma)]. The sample was allowed to dry at room temperature. All the analyses were carried out on an Axima LNR system (Kratos Analytical, Shimadzu) equipped with a nitrogen laser (337 nm). Final spectra were generated within the mass range m/z = 2,000 to 20,000 Da, using the linear mode. *Escherichia coli* DH-5α ribosomal proteins were used for external calibration of the spectra. Microbial identification was performed on the SARAMIS software (AnagnosTec GmbH). The identification was automatically performed by comparison of the ions list of individual samples against the SuperSpectra. Results were considered acceptable for confidence levels ≥ 70%.

### Effect of PD solutions on catheter recovered strains

Four PD solutions formulations were used: three biocompatible solutions: (i) icodextrin [acidic pH, lactate buffered-icodextrin (7.5%)], (ii) bicarbonate/lactate [neutral pH, bicarbonate/lactate buffered-glucose (1.4%)], (iii) bicarbonate [neutral pH, bicarbonate buffered-glucose (1.5%)], and a conventional solution [acidic pH, lactate buffered-glucose (1.5%)] was used as a control.

*P*. *aeruginosa* (PD21.5, 26.4, 30.4, 37.1, 42.4, 50.2, 64.8, 68.7, 82.5, 96.4) and CNS, *S*. *epidermidis* (PD6, 10.2, 11.4, 20.8, 36.1, 45.8, 62.7, 64.6, 85.8, 97), *S*. *caprae/capitis* (PD42.5, 81.1), *S*. *haemolyticus* (PD10.1, 20.7, 50.1) and *S*. *hominis* (PD37.7) isolated in this study were tested. A saline suspension of each strain previously grown in Tryptic Soy Agar (TSA, Liofilchem) at 37°C, 18 h was prepared. The density was adjusted to that of McFarland 0.5 standard and diluted to ~1 x 10^7^ CFU/mL.

Planktonic cells and biofilms were grown as previously described [23]. Ninety-six-wells microtiter plates were conditioned with 150 μL of each PD solution at 37°C, 5% CO_2_ for 24 h. The PD solutions were then aspirated. Standardized bacterial suspensions were added (150 μL/well) and incubated at 37°C, 5% CO_2_ for 24 h. The medium containing planktonic cells was collected and sonicated at 50–60 Hz for 5 min. The biofilms on the wells were washed with saline. Biofilm cells were recovered in 150 µL of TSB-1% (v/v) Tween-20 (Sigma) after 20 min sonication at 50–60 Hz. Viable counts were obtained by serial dilution, and plating on TSA. CFU were enumerated after 24 h at 37°C. Biofilm biomass was determined by the crystal violet assay. Biofilms were stained with 150 μL of crystal violet 10 g/l [1% (w/v) crystal violet (Merck)] for 5 min. The stain was then aspirated and each well was washed twice with ultrapure water, air dried for 20 min and destained with 150 μL of ethanol 100%. Next, 100 μL of the destaining solution was transferred to a new microtiter plate and the absorbance at 570nm (Abs570nm) was recorded (Tecan, Sunrise). TSB and saline were used as positive and negative growth controls, respectively. Two independent assays with four to eight replicates were performed.

### Statistical analyses

Mann-Whitney's test was used for continuous variables and chi-square test with Yates' correction or Fisher's exact test for categorical variables. Whenever appropriate, odds ratio (OR) and 95% confidence intervals (CI) were determined. Heat maps were generated using CIMminer one matrix [24] with the Euclidean distance method and Ward cluster algorithm. Based on the clusters obtained, group comparisons were performed using Mann-Whitney's test or Dunn's multiple comparisons test. P< 0.05 was considered statistically significant. Correlation between the microbial burden and catheter in situ time techniques was determined using linear regression. Statistical analysis was performed using GraphPad Prism.

## Results

### Study population

The PD catheter can be divided in four segments: the external segment (Fig 1Aa)–that is outside the body -, the subcutaneous (Fig 1Ab) and deep cuff (Fig 1Ac)–tunneled segments that transverse the abdominal wall acting as mechanical anchors at two different points–, and the intraperitoneal segment with multiple small openings and the majority of times is coiled (Fig 1Ad)–that is inside the peritoneal cavity. In this study, forty-seven catheters were examined: 16 from patients with infection and 31 from patients without infection (S1 Table). All active patients had a Y-set system and were on neutral pH, low glucose breakdown products—biocompatible solutions. In this population, chronic infections were more likely to be associated with older patients (≥ 55 years old) and a history of previous infection (Table 1).

**Table 1:** Clinic and epidemiologic characteristics of the study population.

Variable	Infection (n = 16)	Absence of infection (n = 31)	Odds ratio [95% confidence interval]	P value
**Age**				
≥ 55 years old	12 (*75*)	13 (*41*.*9*)	4.5 [1.19–17]	0.03
< 55 years old	4 (*25*)	18 (*58*.*1*)		
**Gender**				
Male	11 (*68*.*8*)	19 (*61*.*3*)	1.39 [0.39–5.0]	0.75
Female	5 (*31*.*3*)	12 (*38*.*7*)		
**Comorbidities**				
Diabetes mellitus	4 (*25)*	7 (*22*.*6*)	1.14 [0.28–4.68]	1
No diabetes mellitus	12 (*70*.*6)*	24 (*77*.*4*)		
**Previous RRT**				
PD first	12 (*75*)	21 (*67*.*7*)		
Other^a^	4 (*25*)	10 (*32*.*2*)		
**PD modality**^b^				
APD	5 (*31*.*3*)	15 (*48*.*4*)	0.43 [0.12–1.6]	0.33
CAPD	10 (*62*.*5*)	13 (*41*.*9*)		
**Catheter in situ**				
≥ 50 months	1 (*6*.*3*)	10 (*32*.*3*)	7.62 [0.88–65.84]	0.07
< 50 months	15 (*93*.*8*)	21 (*67*.*7*)		
**History of previous infections**				
Peritonitis				
n≥ 1	10 (*62*.*5*)	8 (*25*.*8*)	4.79 [1.31–17.5]	0.02
n = 0	6 (*37*.*5*)	23 (*74*.*2*)		
Catheter infections				
n≥ 1	7 (*43*.*8*)	4 (*12*.*9*)	5.25 [1.24–22.2]	0.03
n = 0	9 (*56*.*2*)	27 (*87*.*1*)		
**Etiology of ESRD**				
Undetermined	0 (*0*)	7 (22.6)	0.1 [0.005–1.85]	0.08
Others^c^	16 (*100*)	24 (77.4)		

^a,^ hemodialysis and renal transplant;

^b,^ 1 patient in the group “Infection” and 3 patients in the group “Absence of infection” were not active/ have not initiated the therapy;

^c,^ Diabetic nephropathy, systemic disease, glomerulonephritis, autosomal dominant polycystic kidney disease, interstitial nephritis, hereditary nephropathy/malformation. Values are numbers with percentages in parenthesis.

PD, peritoneal dialysis, APD, automatic PD; CAPD, continuous ambulatory PD; ESRD, end-stage renal disease; RRT, renal replacement therapy.

### Characteristics of biofilms on PD catheters

**Fig 2:**
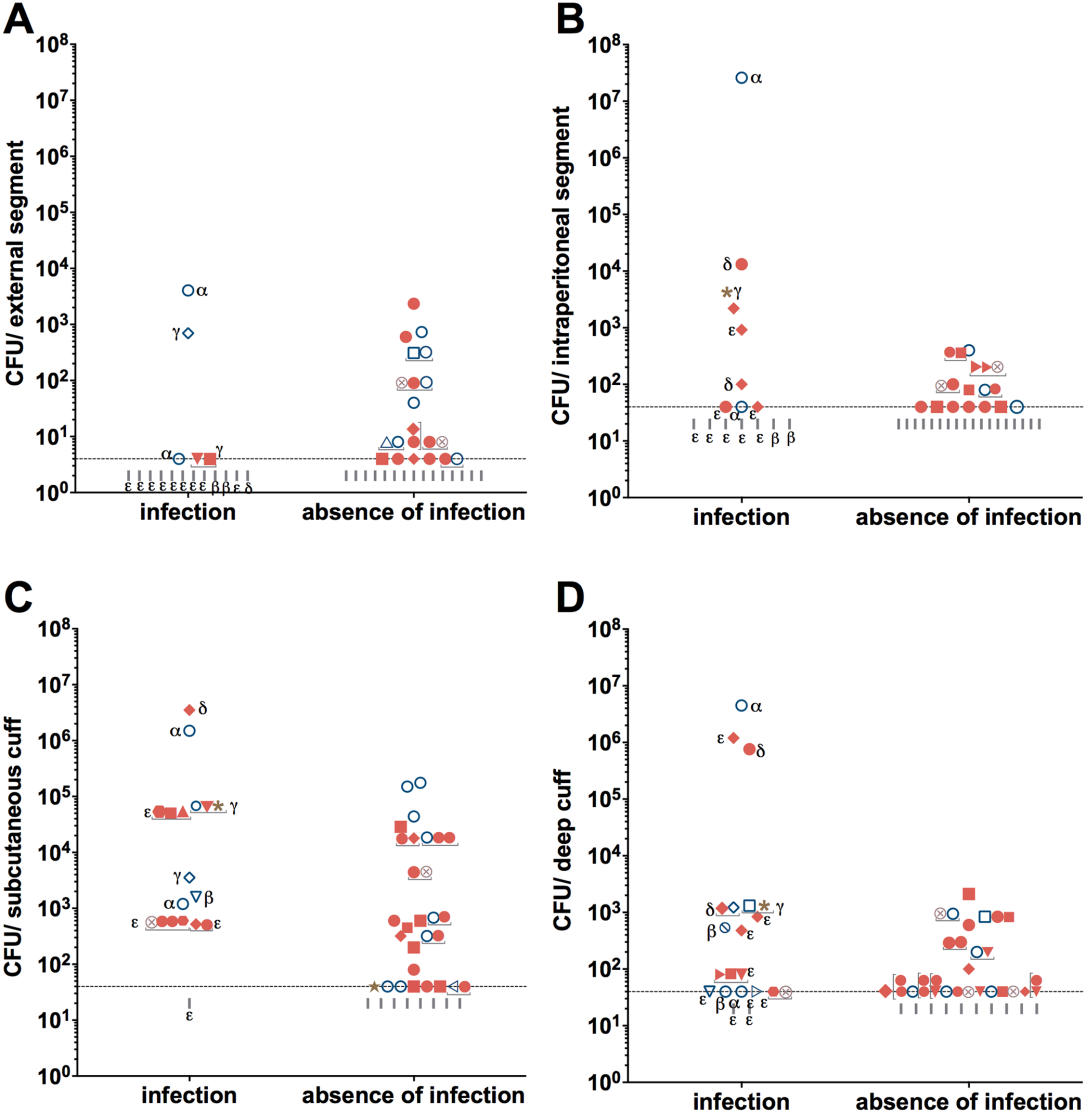
Microbial yield on PD catheters external (A) and intraperitoneal (B) segments and subcutaneous (C) and deep (D) cuffs in patients with and without infection. Data is presented as colony-forming units (CFU)/segment with the corresponding microorganisms represented by specific symbols. dot, coagulase negative Staphylococci (including Staphylococcus epidermidis, S. haemolyticus, S. caprae/ capitis, S. hominis, S. auricularis); diamond filled, *Staphylococcus aureus*; square filled, Corynebacterium spp.; down-pointing triangle filled, Micrococcus luteus; up-pointing triangle filled, Enterococcus faecalis; hexagon filled, Streptococcus spp.; right-pointing triangle filled, Bacillus spp.; circle, Pseudomonas *aeruginosa*; square, Sphingomonas spp.; diamond, Alcaligenes faecalis; down-pointing triangle, Serratia marcescens; up-pointing triangle, Burkholderia sp.; left-pointing triangle, Stenotrophomonas maltophilia; right-pointing triangle, Escherichia coli; crossed out circle, Enterobacter aerogenes; star, Candida parapsilosis; asterisk, Candida glabrata; circle quartered, non-identified microorganism. Absence of microorganisms is represented by, vertical line. Polymicrobial cultures are identified with a half tick-up line. In the group “infection” the cause of removal is indicated as alpha, for refractory peritonitis; beta, for relapsing peritonitis; gamma, fungal peritonitis; delta, for catheter-related peritonitis and epsilon, for chronic catheter infections. The dashed line indicates the culture method detection limit.

The literature suggests that the colonization differs among the different segments (e.g. [25]). Therefore, in this study we analysed each segment separately. Catheters culture was positive for any segment in 87.5% (n = 14) of those removed from patients with infection and in 90.3% (n = 28) without infection (OR, 0.75; CI, 0.11–5.02; P = 1). Furthermore, the distribution of positive cultures along the catheter was similar in both groups (Fig 1B, P = 0.72). However, the analyses of the microbial yield revealed that the colonization level in the cuffs (Fig 2C and 2D) is higher in catheters removed due to infectious vs. non-infectious causes: 2600 vs. 60 CFU/subcutaneous cuff and 280 vs. 40 CFU/deep cuff (infection vs. absence of infection, P = 0.01; S2 Table). Notably, in both groups the median microbial yield was higher in the cuffs (Fig 2C and 2D) than in the silicone segments (Fig 2A and 2B): infection group, 720 CFU/cuffs vs. 20 CFU/silicone segment, P< 0.0001; and absence of infection group, 40 CFU/cuffs vs. 20 CFU/silicone segment, P< 0.0001], thus pointing to subclinical colonization of the cuffs. In this population there was no correlation between microbial burden and catheter in situ time (S1 Fig).

However, we noticed that in each group the microbial species recovered from the cuffs and silicone segments were similar (S3 Table). Therefore, we considered the overall microbial species recovered from the catheters (Fig 2). In catheters removed due to infectious causes Staphylococci and *P*. *aeruginosa* were the predominant species (32% and 20%, respectively) followed by *Corynebacterium* spp. = *Micrococcus luteus = Streptococcus* spp. = *Alcaligenes faecalis* = *Candida glabrata* > *S*. *marcescens* > *Enterococcus faecalis* = *Bacillus* spp. = *Sphingomonas* spp. = *Enterobacter aerogenes* = *Escherichia coli*. In 75% of the catheters (n = 12) the infectious causing agent was recovered, although in 25% of these (n = 3), together with other microorganisms. However, in the case of two patients with *S*. *aureus* and *S*. *xylosus* chronic catheter infection, other microorganisms were found while in two patients with *Corynebacterium* spp. and *P*. *aeruginosa* chronic catheter infections, no microorganisms were recovered.

Notably, in catheters removed due to non-infectious causes Staphylococci and *P*. *aeruginosa* were also the predominant species (43.3% and 22.7%, respectively) but then followed by *Corynebacterium* spp. > *M*. *luteus* > *Bacillus* spp. = *Sphingomonas* spp. > *Burkholderia* sp. = *Stenotrophomonas maltophilia* = *C*. *parapsilosis*.

We also analysed the proportion of the different species found in each catheter (S4 Table). Curiously, *P*. *aeruginosa* and CNS were only co-isolated in the same catheter in the group of patients without infection, where *P*. *aeruginosa* was the dominant species in 6 out of these 9 microbial communities. The presence of these microorganisms was less likely to be associated with catheters removed due to infectious causes than non-infectious ones (OR, 0.22; CI, 0.08–0.62; P = 0.005 and OR, 0.22; CI, 0.08–0.62; P = 0.045, respectively), again pointing to a subclinical colonization with these menacing agents.

### Impact of PD solutions on microorganisms

Due to the relevance of CNS and *P*. *aeruginosa* as colonizers of PD catheters, their involvement in recalcitrant infections [10–13] and their known ability to form biofilms [26], it is important to elucidate how dialytic factors affect their survival. Therefore, the effect of three biocompatible PD solutions being used by the patients (biocompatible bicarbonate, bicarbonate/lactate and icodextrin solutions) was tested on ten *P*. *aeruginosa* and 16 CNS (10 *S*. *epidermidis*, 2 *S*. *caprae/capitis*, 3 *S*. *haemolyticus* and 1 *S*. *hominis*) strains previously recovered from the catheters. A conventional solution was used as a control.

**Fig 3:**
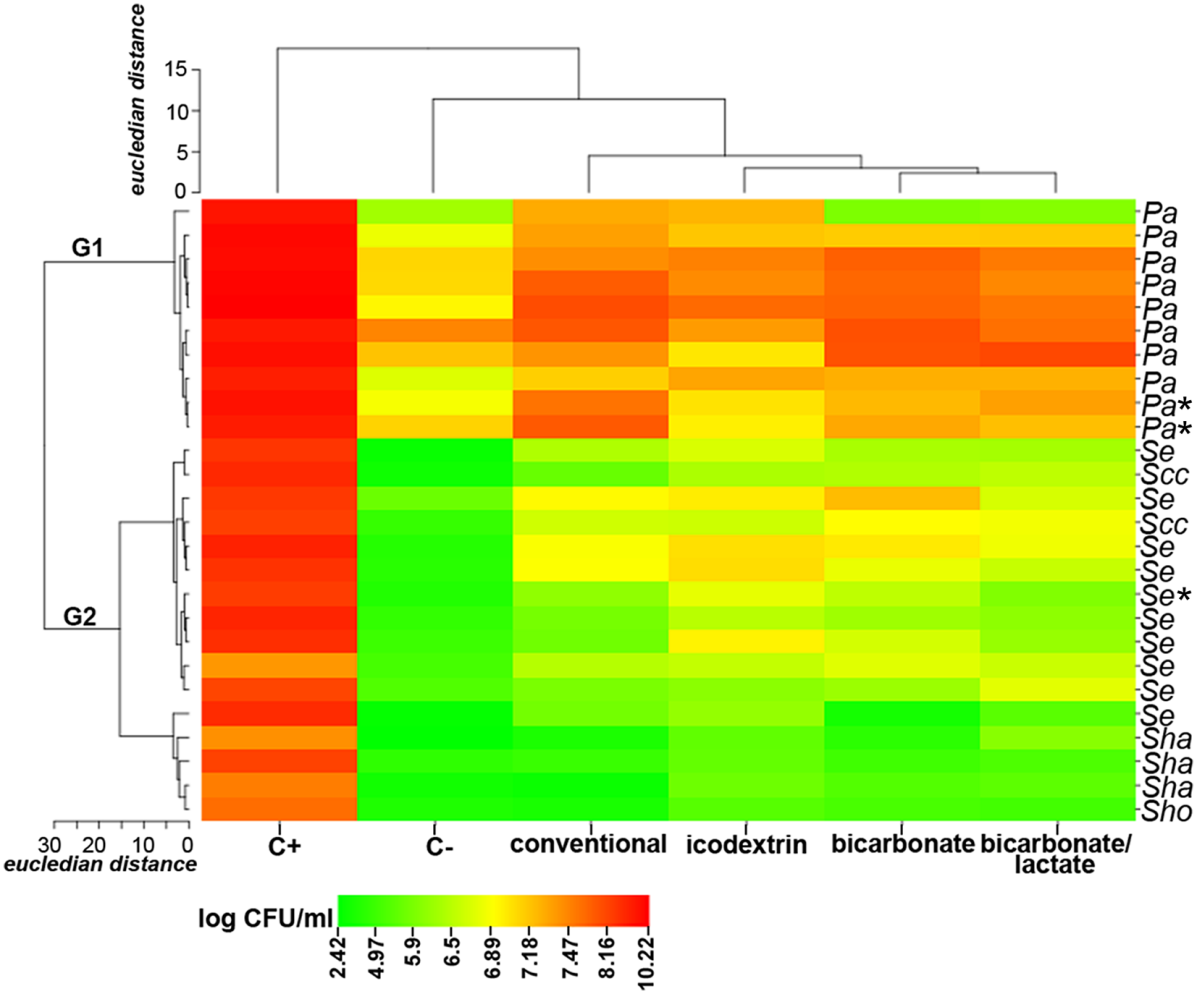
Effect of PD solutions on *P. aeruginosa* and CNS planktonic cells. Mean log CFU/mL after 24 h exposure to PD solutions was used for the generation of the heat map. The strains are indicated on the right (Pa, P. aeruginosa; Se, S. epidermidis, Scc, S. caprae/capitis; Sha, S. haemolyticus; Sho, S. hominis). Strains derived from catheters of patients with infection are indicated with an asterisk. Main strains clusters are identified as group 1 (G1) and group 2 (G2). The conventional and biocompatible (bicarbonate, bicarbonate/lactate, icodextrin) PD solutions are indicated on the bottom, as well as the growth controls: positive (C+, TSB culture medium) and negative (C-, saline). The initial inoculum concentration was of ~1 × 107 CFU/mL (7 log CFU/mL). Dendrograms across the top and left of the heat map show the relationship between PD solutions and strains, respectively. Distance values are depicted by the gradient colour ranging from green (lowest value) to red (highest value).

Firstly, we evaluated the effect on planktonic cells (Fig 3, S5 Table). The strains were resolved in two major groups (Fig 3) comprising *P*. *aeruginosa* (**G1**) and CNS (**G2**) according to similarities in their growth profile. PD solutions inhibited the growth of CNS strains that invariably exhibited significantly lower number of culturable cells vs. the initial inoculum. In contrast, a less detrimental effect was observed on *P*. *aeruginosa* strains that did not grow in the presence of bicarbonate and bicarbonate/lactate solutions but exhibited slightly higher cell numbers in conventional and icodextrin solutions. Overall, *P*. *aeruginosa* isolates displayed higher cell densities than CNS after exposure to the solutions (Fig 3; P< 0.0001). Notably, conventional and biocompatible solutions had a similar effect on planktonic cells (P> 0.05).

**Fig 4:**
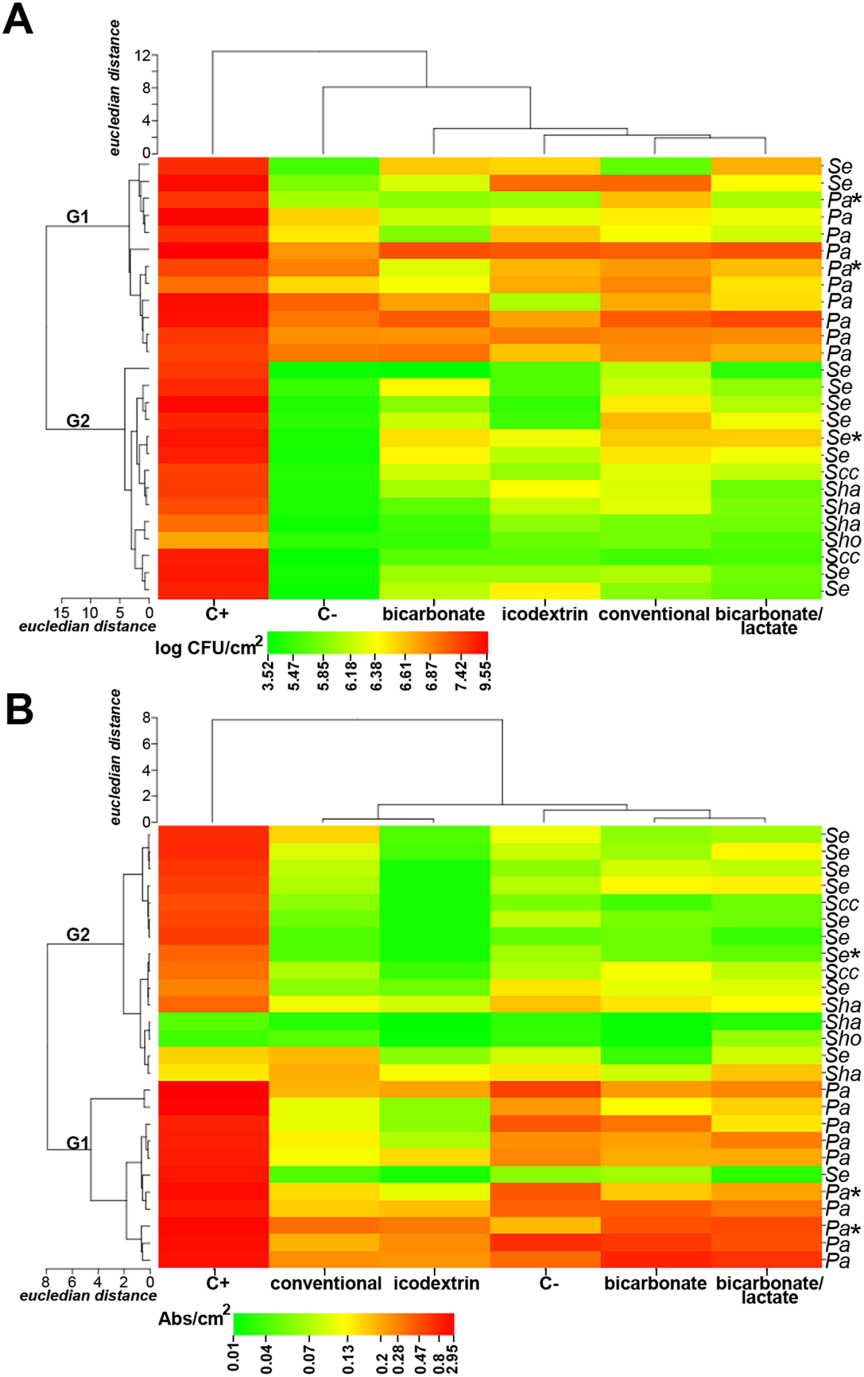
Effect of PD solutions on *P. aeruginosa* and CNS biofilm cells (A) and biomass, cells and extracellular matrix (B). Mean log CFU/cm2 (A) and Abs570nm/cm2 (B) after 24 h exposure to PD solutions were used for the generation of the heat maps. The strains are indicated on the right (Pa, *P. aeruginosa*; Se, S. epidermidis, Scc, S. caprae/capitis; Sha, S. haemolyticus; Sho, S. hominis). Strains derived from catheters of patients with infection are indicated with an asterisk. Main strains clusters are identified as group 1 (G1) and group 2 (G2). The conventional and biocompatible (bicarbonate, bicarbonate/lactate, icodextrin) PD solutions are indicated on the bottom, as well as the growth controls: positive (C+, TSB culture medium) and negative (C-, saline). Dendrograms across the top and left of the heat map show the relationship between PD solutions and strains, respectively. Distance values are depicted by the gradient colour ranging from green (lowest value) to red (highest value).

To determine the effect of PD solutions on biofilms, cell density (Fig 4A, S5 Table) and whole biofilm biomass (Fig 4B, S5 Table) were evaluated. In the presence of PD solutions all the strains formed biofilm with ≥ 3 × 10^3^ CFU/cm^2^ (Fig 4A) and biomass ≥ 0.01 Abs570/cm^2^ (Fig 4B), although in a less extent than in the positive control. However, *P*. *aeruginosa* exhibited significantly higher microbial yields and biomass (Fig 4, G1) than CNS strains (Fig 4, G2). In general, conventional and biocompatible solutions elicited a similar effect on strains biofilm formation. Nevertheless, strains grown in icodextrin exhibited less biofilm biomass than in bicarbonate/lactate solutions (P = 0.03), mainly driven by the negative impact of the former solution on CNS biomass [0.02 (interquartile range, IQR, 0.02–0.04) vs. 0.07 (IQR, 0.05–0.11) Abs570nm/cm^2^, P = 0.008 for CNS; 0.14 (IQR, 0.05–0.23) vs. 0.27 (IQR, 0.15–0.47) Abs570nm/cm^2^ for *P*. *aeruginosa*; icodextrin vs. bicarbonate/lactate].

## Discussion

PD infections management is focused on the elimination of planktonic microorganisms expected to respond to local antimicrobial therapies. However, a significant amount of cases do not resolve and require catheter removal [17]. From other fields [27] it is anticipated that biofilms on catheters threaten the resolution of infections. The recent guidelines for the diagnosis and treatment of biofilm infections [27] do not make recommendations for PD patients, demanding a deeper investigation.

Using ex vivo and in vitro microbiological approaches we show that biofilms, highly dominated by Staphylococci and *P*. *aeruginosa*, are ubiquitous on PD catheters, although their microbial burden may allow the discrimination of an infective status. This widespread incidence is supported by PD solutions.

The long-term implantation of the catheter, multiple sources of potential contamination including manipulation by the patient and the moist environment provide unique conditions for the establishment of biofilm. We report the presence of culturable microorganisms at the surfaces of PD catheters (Fig 1) despite the absence of infection, as previously observed. Even though, in some cases we did not recover microorganisms from the catheter segments, similarly to the observed in catheters removed from patients with or without infection [15]. This can be due to the presence of unculturable microorganisms, non ability of certain microorganisms to grow under the experimental conditions used, or to the undergoing antimicrobial therapy, in the cases of established infection. Therefore, future studies may include a multi-modal approach that also includes molecular-based and microscopy approaches [28].

CNS and *P*. *aeruginosa*—major threats in PD chronic infections [10–13]—were the dominant microbial species recovered in both groups (Fig 2), in accordance with previous reports [15]. This suggests that contamination and exit-site/tunnel infections are major players for microbial entry. It should be noted that the majority of previously studies were performed on catheters recovered between 1990 and 2000. After that period PD technique and preventive protocols have changed in parallel with the spectrum of infection causing agents [29]. To the best of our knowledge, we firstly report the recovery of *A*. *faecalis*, *Bacillus* spp. and *S*. *maltophilia* from PD catheters. While *A*. *faecalis* was recovered from the catheter of a patient with an infection caused by this microorganism, the other two microbial species were recovered from catheters of patients without infection. This is particularly relevant because these species are associated with poor prognosis and loss of catheter [30, 31]. Furthermore, it is particularly interesting that in five catheters removed due to infection other microorganisms besides the infection agent were found. This strongly suggests a biofilm community supporting the co-existence of several species contributing to the persistence of the infection, and the need to further explore the interactions between the different microorganisms, even in the subclinical infections.

However, our study is unique in that it compares the microbial density in PD catheters removed due to infectious and non-infectious causes, while a previous study only reported burden estimates (number of colonies when up to 100 and moderate growth –10^5^–10^6^ CFU/mL—and heavy pure growth—10^7^–10^9^ CFU/mL) in catheters removed from patients with infection [25]. We found that microbial burdens are higher on cuffs than silicone segments in the presence of infection. Indeed, in hemodialysis catheters thicker and highly colonized biofilms have been associated with infection [32, 33]. Importantly, we show that cuffs are common and significant reservoirs of microorganisms in subclinical settings. This is in line with previous observations of colonization [34] and persistence of bacteria [25] on the cuffs. However, it cannot be disregarded that the catheter removal cause, initiated antimicrobial treatment and microorganisms’ migration along the catheter can be confounding variables. While the same methodology was used through all the study, the microbiological analysis is semi-quantitative, as the complete recovery of microorganisms from the catheter cannot be assured and some of the microorganisms may be unculturable.

We took advantage of the cohort of microorganisms recovered from PD catheters to study the short-term effect of biocompatible solutions. Biocompatible solutions are increasingly used due to promising benefits on the peritoneal membrane [19] but, to date their role on biofilms is not known. Using in vitro studies, we found that biocompatible solutions restrain the growth of PD strains in suspension and, as firstly shown here, support biofilm formation, similarly to conventional solutions (Figs 3 and 4). Icodextrin reduced biofilm biomass vs. bicarbonate/lactate solution, probably due to the lack of glucose as a carbon source in the former solution. However, this may be overcome by the patient dialysate composition changes that occur during the day, as described for *S*. *epidermidis* with conventional solutions [35]. These results seem to meet the above-mentioned observations of PD solutions similar outcome on patients’ infection rates [20]. It was also interesting to find that PD solutions have a more detrimental effect on CNS than *P*. *aeruginosa* strains, what may at least in part underlie the clinical observations that CNS infections tend to be more benign than *P*. *aeruginosa* ones [36, 37]. To get further insights into the role of microorganisms in infections, it would be interesting to understand the biofilm forming ability of the strains responsible for the infectious episodes in comparison with the ones isolates from non-infectious ones in the presence of PD solutions.

These findings suggest that although the biofilm on PD catheter, per se, do not result in infection, when it reaches a certain density, the patient immune defenses may be overcome promoting the (re)infection.

In terms of diagnosis, our study calls for biofilm in vivo visualization techniques and establishment of infectivity cut-offs. Furthermore, we observed that a previous infection was associated with high risk of chronic infection (Table 1), suggesting inefficient microorganisms removal or changes in imunomodulatory pathways, predisposing to infection, as previously anticipated [38]. Therefore, controlled trials that include the assessment of biofilm-associated immune biomarkers are required, besides the already established infection fingerprints [39].

In terms of treatment, it seems critical to consider the growth and susceptibility of microorganisms on biofilms. This can be achieved estimating the antimicrobial tolerance of biofilm cells and using it instead of the routine minimal inhibitory concentrations. Although the use of oral/systemic antimicrobials in PD catheter exit-site management is debated [17], the high colonization rates at the cuffs observed herein suggest the need of more aggressive treatments to overcome the risk of acute-to-chronic infection progression.

In terms of preventive strategies, the high predominance of CNS and *P*. *aeruginosa* emphasizes the importance of patient training towards aseptic technique of the catheter transfer set [22] and exit-sites. This should be supported by the development of new catheters that promote better integration in the abdominal wall. Additionally, strategies aimed at reducing the density or at resensitising microorganisms on biofilms, should be investigated. Current evidences do not provide clear benefits of heparin, urokinase, tissue plasminogen activator in chronic PD infections treatment [17]. However, these and emerging adjunctive therapies, as DNase [40], can be tested as infection preventive strategies.

Our results show that microbial biofilms are a common feature in PD catheters, whose extent increases in the presence of infection. This provides insights into the pathogenesis of chronic PD infections and the foundation of future studies and approaches.

## Supporting Information

S1 Fig Correlation between the catheter microbial burden and in situ time in the group in which the catheter was removed due to infectious causes (A) and non-infectious causes (B). Each dot represents a patient.
(PDF)

S1 Table Catheter removal causes stratification. (PDF)

S2 Table Microbial yield in specific catheter segments. (PDF)

S3 Table Type of microorganisms isolated from specific catheter segments within each group. (PDF)

S4 Table Ratio of microbial species recovered in each catheter. (PDF)

S5 Table Effect of PD solutions on planktonic cells and biofilms of strains recovered in this study. (PDF)
