# Supplementary material for: Deciphering the Contribution of Biofilm to the Pathogenesis of Peritoneal Dialysis Infections: Characterization and Microbial Behaviour on Dialysis Fluids
Source: PLoS One. 2016 Jun 23;11(6):e0157870. doi: 10.1371/journal.pone.0157870 (PMC4918928; doi:10.1371/journal.pone.0157870)
Supplement: S1 Fig — Each dot represents a patient. (PDF) [file pone.0157870.s001.pdf]

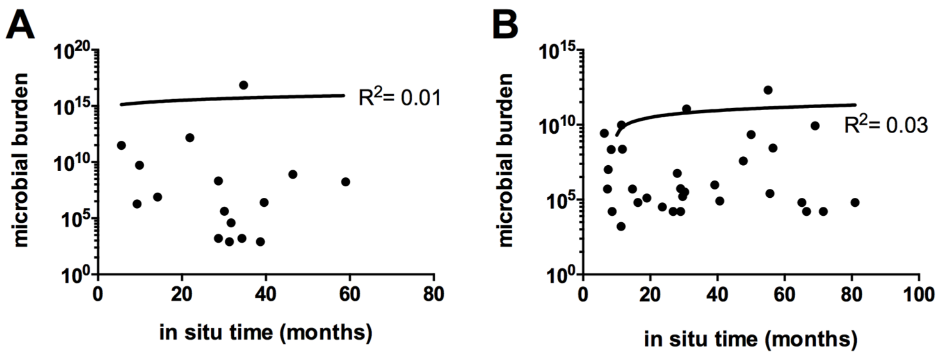

S1 Fig. Correlation between the catheter microbial burden and in situ time in the group in which the catheter was removed due to infectious causes **(A)** and non-infectious causes **(B)**. Each dot represents a patient.
