# Supplementary material for: Deciphering the Contribution of Biofilm to the Pathogenesis of Peritoneal Dialysis Infections: Characterization and Microbial Behaviour on Dialysis Fluids
Source: PLoS One. 2016 Jun 23;11(6):e0157870. doi: 10.1371/journal.pone.0157870 (PMC4918928; doi:10.1371/journal.pone.0157870)
Supplement: S1 Table — (PDF) [file pone.0157870.s002.pdf]

S1 Table. Catheter removal causes stratification

**Infection**			**Absence of infection**	
	(n)	infectious agent		(n)
Refractory peritonitis	2	*Pseudomonas aeruginosa*	Renal transplant	17
Relapsing peritonitis	1	*Serratia marcescens*	Catheter dysfunction[a]	2
	1	*Enterobacter aerogenes*	Recovery of function	2
Fungal peritonitis	1	*Candida glabrata*	Loss of ultrafiltration	3
Catheter-related peritonitis	1	*Staphylococcus aureus*/ *Alcaligenes faecalis*	Non-adhesion/adaptation to PD	7
	1	*Staphylococcus haemolyticus*		
Chronic catheter infections	4	*Staphylococcus aureus*		
	1	*Escherichia coli*		
	1	*Corynebacterium*		
	1	*Staphylococcus xylosus*		
	2	*Pseudomonas aeruginosa*		

Values are number of catheters. PD, peritoneal dialysis. [a], catheter malfunction, malposition or migration, without the presence of any clinical and analytical sign of infection.
