# Supplementary material for: Deciphering the Contribution of Biofilm to the Pathogenesis of Peritoneal Dialysis Infections: Characterization and Microbial Behaviour on Dialysis Fluids
Source: PLoS One. 2016 Jun 23;11(6):e0157870. doi: 10.1371/journal.pone.0157870 (PMC4918928; doi:10.1371/journal.pone.0157870)
Supplement: S2 Table — (PDF) [file pone.0157870.s003.pdf]

S2 Table. Microbial yield in specific catheter segments

**Segment**	**Infection** (CFU/segment)	**Absence of infection** (CFU/segment)	**P value**
External segment	2 [2 - 3.5]	2 [2 - 8]	0.18
Subcutaneous cuff	2.6×10^3 [5.8×10^2 - 4.3×10^5]	6×10^1 [2×10^1 - 1.6×10^3]	0.01
Deep cuff	2.8×10^2 [4×10^1 - 1.3×10^3]	4×10^1 [2×10^1 – 5.5×10^1]	0.01
Intraperitoneal segment	4×10^1 [2×10^1 – 1.9×10^3]	2×10^1 [2×10^1 – 4×10^1]	0.13

Values are median [interquartile range]. P values calculation based on two-tailed Mann Whitney test.
