# Supplementary material for: Deciphering the Contribution of Biofilm to the Pathogenesis of Peritoneal Dialysis Infections: Characterization and Microbial Behaviour on Dialysis Fluids
Source: PLoS One. 2016 Jun 23;11(6):e0157870. doi: 10.1371/journal.pone.0157870 (PMC4918928; doi:10.1371/journal.pone.0157870)
Supplement: S3 Table — (PDF) [file pone.0157870.s004.pdf]

S3 Table. Type of microorganisms isolated from specific catheter segments within each group

**Group/ Microorganism**	Cuffs[a]	Silicone[b]	**Odds ratio [95% confidence interval]**	**P value**
**Infection**				
**Type**[c †]				
Gram positive	19 (*52.8*)	8 (*57.1*)	-	0.98[e]
Gram negative	13 (*36.1*)	5 (*35.7*)		
Fungi	2 (*5.6*)	1 (*7.1*)		
**Gram-positive species**[d]				
CNS*	4 (*21.1*)	2 (*25*)	0.8 [0.11 – 5.59]	1[f]
all other Gram positive spp.[§]	15 (*78.9*)	6 (*75*)		
**Gram-negative species**[d]				
Pseudomonas aeruginosa	6 (*46.2*)	4 (*80*)	0.21 [0.02 – 2.48]	0.31[f]
all other Gram negative spp.[¶]	7 (*53.8*)	1 (*20*)		
**Absence of infection**				
**Type**[c††]				
Gram positive	38 (*65.5*)	24 (*61.5*)	-	0.68[e]
Gram negative	15 (*25.9*)	11 (*28.2*)		
Fungi	1 (*1.7*)	0 (*0*)		
**Gram-positive species**[d]				
CNS*	20 (*52.6*)	15 (*62.5*)	0.67 [0.23 – 1.89]	0.60[f]
all other Gram positive spp.[§]	18 (*47.4*)	9 (*37.5*)		
**Gram-negative species**[d]				
Pseudomonas aeruginosa	13 (*86.7*)	9 (*81.8*)	1.44 [0.17 – 12.24]	1[f]
all other Gram negative spp.[¶]	2 (*13.3*)	2 (*18.2*)		

-, not applicable; [a], subcutaneous and deep cuff; [b], external and intraperitoneal segments; Values are number of observations of each microbial species with percentages in italic within parenthesis, with respect to [c], all the microorganisms isolated, or [d] microorganisms from a specific type. P value calculation based on [e], Chi-square or [f], Fisher's exact test. Microorganisms not identified: [†] 2 in the cuffs, [††] 4, in each segment. *, CNS, coagulase negative Staphylococci, including *Staphylococcus epidermidis*, S. haemolyticus, S. caprae/ capitis, S. hominis, S. auricularis; [§], comprising *Staphylococcus aureus*, *Corynebacterium* spp., *Micrococcus luteus*, *Enterococcus faecalis*, *Streptococcus* spp. and *Bacillus* spp.; [¶], comprising *Sphingomonas* spp., *Alcaligenes faecalis*, *Serratia marcescens*, *Burkholderia* sp., *Stenotrophomonas maltophilia*, *Escherichia coli* and *Enterobacter aerogenes*
