# Supplementary material for: Deciphering the Contribution of Biofilm to the Pathogenesis of Peritoneal Dialysis Infections: Characterization and Microbial Behaviour on Dialysis Fluids
Source: PLoS One. 2016 Jun 23;11(6):e0157870. doi: 10.1371/journal.pone.0157870 (PMC4918928; doi:10.1371/journal.pone.0157870)
Supplement: S4 Table — (PDF) [file pone.0157870.s005.pdf]

S4 Table. Ratio of microbial species recovered in each catheter

	**Sp.**	**ratio**	**Sp.**	**ratio**	**Sp.**	**ratio**	**Sp.**	**ratio**	**Sp.**	**ratio**
**Infection**										
**ID**										
**1**	*P. aeruginosa*	1	-	-	-	-	-	-	-	-
**2**	*A. faecalis*	4	*S. aureus*	1	-	-	-	-	-	-
**3**	*S. aureus*	1	-	-	-	-	-	-	-	-
**4**	*Streptococcus spp.*	303	*S. epidermidis*	21	*n.i.*	21	*S. haemolyticus*	1		
**5**	*E. coli*	1	-	-	-	-	-	-	-	-
**6**	*Streptococcus spp.*	2727	*E. faecalis*	212	*Corynebacterium spp.*	89	*Bacillus spp.*	1	*M. luteus*	1
**7**	*S. aureus*	5	*S. haemolyticus*	1	-	-	-	-	-	-
**8**	*P. aeruginosa*	1	-	-	-	-	-	-	-	-
**9**	*S. marcescens*	1	-	-	-	-	-	-	-	-
**10**	*S. aureus*	3	*S. caprae/ capitis*	1	-	-	-	-	-	-
**11**	*P. aeruginosa*	65714	*C. glabrata*	137	*M. luteus*	39	*Sphingomonas spp.*	1	*Corynebacterium spp*	1
**12**	*P. aeruginosa*	1	-	-	-	-	-	-	-	-
**13**	*E. aerogenes*	1	-	-	-	-	-	-	-	-
**14**	*S. aureus*	1	-	-	-	-	-	-	-	-
**No Infection**										
**ID**										
**1**	*Corynebacterium spp.*	1	-	-	-	-	-	-	-	-
**2**	*S. epidermidis*	1	-	-	-	-	-	-	-	-
**3**	*M. luteus*	1	-	-	-	-	-	-	-	-
**4**	*S. haemolyticus*	99	*S. epidermidis*	1	-	-	-	-	-	-
**5**	*S. epidermidis*	1	-	-	-	-	-	-	-	-
**6**	*S. epidermidis*	210	*P. aeruginosa*	117	*S. caprae/ capitis*	3	n.i.	3	*S. haemolyticus*	1
**7**	*Corynebacterium spp.*	30	*Sphingomonas spp.*	11	*P. aeruginosa*	8	*S. caprae/ capitis*	1		

**8**	*P. aeruginosa*	999	n.i.	1	-	-	-	-	-	-
**9**	*S. aureus*	1	-	-	-	-	-	-	-	-
**10**	*P. aeruginosa*	30	*S. hominis*	4	*S. epidermidis*	1	n.i.	1	-	-
**11**	*Corynebacterium spp.*	1	-	-	-	-	-	-	-	-
**12**	*S. epidermidis*	20	*P. aeruginosa*	3	*S. hominis*	1	*S. haemolyticus*	1	-	-
**13**	*P. aeruginosa*	9091	*S. epidermidis*	2	*M. luteus*	1	-	-	-	-
**14**	*C. parapsilosis*	10	*S. aureus*	1	-	-	-	-	-	-
**15**	*P. aeruginosa*	5	*S. epidermidis*	1	-	-	-	-	-	-
**16**	*Bacillus spp.*	4	*Corynebacterium spp.*	1	n.i.	1	-	-	-	-
**17**	*M. luteus*	1	-	-	-	-	-	-	-	-
**18**	*M. luteus*	19	*S. epidermidis*	1	-	-	-	-	-	-
**19**	n.i.	1	-	-	-	-	-	-	-	-
**20**	*Corynebacterium spp.*	2	n.i.	1	-	-	-	-	-	-
**21**	*Corynebacterium spp.*	1	-	-	-	-	-	-	-	-
**22**	*P. aeruginosa*	2	*S. epidermidis*	1	-	-	-	-	-	-
**23**	*P. aeruginosa*	10988	*S. caprae/ capitis*	1	n.i.	1	-	-	-	-
**24**	*S. epidermidis*	3	*S. maltophilia*	1	-	-	-	-	-	-
**25**	*S. aureus*	4	*S. epidermidis*	1	-	-	-	-	-	-
**26**	*S. epidermidis*	47	*P. aeruginosa*	3	*Burkholderia* sp.	1	-	-	-	-
**27**	n.i.	76	*Corynebacterium spp.*	18	*S. epidermidis*	4	*Sphingomonas spp.*	1	-	-
**28**	*S. epidermidis*	1	-	-	-	-	-	-	-	-

The values represent the proportion of different microbial species cultured from the catheter based on the number of CFU recovered. For example, in the Infection group, catheter #1, only *P. aeruginosa* was recovered; but in catheter #2 for each *S. aureus* CFU there were 4 *A. faecalis* CFU.
