# Supplementary material for: Deciphering the Contribution of Biofilm to the Pathogenesis of Peritoneal Dialysis Infections: Characterization and Microbial Behaviour on Dialysis Fluids
Source: PLoS One. 2016 Jun 23;11(6):e0157870. doi: 10.1371/journal.pone.0157870 (PMC4918928; doi:10.1371/journal.pone.0157870)
Supplement: S5 Table — (PDF) [file pone.0157870.s006.pdf]

S5 Table. Effect of PD solutions on planktonic cells and biofilms of strains recovered in this study

	**Planktonic Culturable cells (log CFU/mL)**						**Biofilm Culturable cells (log CFU/cm^2)**						**Biomass (Abs570nm/cm^2)**					
	**C+**	**C-**	**conventional**	**bicarbonate**	**bicarbonate/lactate**	**icodextrin**	**C+**	**C-**	**conventional**	**bicarbonate**	**bicarbonate/lactate**	**icodextrin**	**C+**	**C-**	**conventional**	**bicarbonate**	**bicarbonate/lactate**	**icodextrin**
Pa PD21.5	9.12 (*0.25*)	6.75 (*0.36*)	7.12 (*0.13*)	7.26 (*0.19*)	7.26 (*0.23*)	7.28 (*0.28*)	8.25 (*0.3*)	6.54 (*0.15*)	6.21 (*0.3*)	6.29 (*0.45*)	6.29 (*0.18*)	6.44 (*0.16*)	2.95 (*0.62*)	0.57 (*0.2*)	0.21 (*0.07*)	0.2 (*0.15*)	0.27 (*0.09*)	0.18 (*0.07*)
Pa PD26.4*	9.41 (*0.18*)	6.86 (*0.25*)	7.52 (*0.4*)	7.23 (*0.3*)	7.29 (*0.3*)	7.07 (*0.29*)	7.38 (*0.21*)	6.87 (*0.26*)	6.27 (*0.29*)	6.67 (*0.15*)	6.61 (*0.2*)	6.75 (*0.32*)	1.31 (*0.36*)	0.42 (*0.09*)	0.16 (*0.14*)	0.10 (*0.05*)	0.21 (*0.08*)	0.14 (*0.07*)
Pa PD30.4	9.88 (*0.20*)	7.1 (*0.20*)	7.35 (*0.21*)	7.59 (*0.19*)	7.50 (*0.34*)	7.43 (*0.24*)	9.55 (*0.2*)	6.77 (*0.2*)	7.26 (*0.19*)	7.1 (*0.24*)	7.17 (*0.34*)	7.02 (*0.21*)	1.31 (*0.3*)	0.79 (*0.14*)	0.61 (*0.06*)	0.26 (*0.05*)	0.47 (*0.11*)	0.18 (*0.09*)
Pa PD37.1	10 (*0.40*)	6.82 (*0.39*)	7.29 (*0.37*)	7.12 (*0.32*)	7.13 (*0.19*)	7.14 (*0.16*)	7.49 (*0.25*)	6.79 (*0.16*)	6.78 (*0.24*)	6.89 (*0.22*)	6.83 (*0.33*)	6.84 (*0.13*)	1.15 (*0.18*)	0.38 (*0.03*)	0.39 (*0.03*)	0.17 (*0.07*)	0.3 (*0.05*)	0.16 (*0.05*)
Pa PD42.4	9.29 (*0.28*)	7.4 (*0.21*)	7.66 (*0.33*)	7.73 (*0.43*)	7.52 (*0.27*)	7.30 (*0.18*)	7.97 (*0.23*)	6.9 (*0.12*)	7.09 (*0.21*)	6.73 (*0.16*)	7.31 (*0.3*)	7.07 (*0.4*)	0.87 (*0.11*)	0.24 (*0.03*)	0.21 (*0.06*)	0.07 (*0.04*)	0.28 (*0.12*)	0.13 (*0.04*)
Pa PD50.2	9.35 (*0.26*)	6.43 (*0.5*)	7.27 (*0.27*)	5.95 (*0.37*)	6.11 (*0.30*	7.23 (*0.28*)	7.41 (*0.18*)	6.89 (*0.18*)	6.93 (*0.4*)	6.61 (*0.12*)	6.69 (*0.35*)	6.8 (*0.22*)	1.29 (*0.2*)	0.36 (*0.04*)	0.83 (*0.11*)	0.23 (*0.14*)	0.79 (*0.16*)	0.23 (*0.07*)
Pa PD64.8	10.09 (*0.11*)	7.09 (*0.13*)	7.59 (*0.18*)	7.54 (*0.28*)	7.36 (*0.06*)	7.36 (*0.06*)	8.01 (*0.26*)	7.02 (*0.34*)	6.73 (*0.16*)	6.12 (*0.16*)	6.52 (*0.09*)	6.71 (*0.07*)	0.84 (*0.17*)	0.42 (*0.02*)	0.3 (*0.04*)	0.05 (*0.05*)	0.13 (*0.05*)	0.10 (*0.04*)
Pa PD68.7	9.7 (*0.2*)	7.18 (*0.23*)	7.33 (*0.28*)	7.72 (*0.12*)	7.85 (*0.17*)	7.07 (*0.14*)	7.70 (*0.34*)	6.48 (*0.19*)	5.85 (*0.37*)	6.59 (*0.61*)	6.24 (*0.22*)	6.36 (*0.24*)	2.80 (*0.09*)	0.22 (*0.04*)	0.12 (*0.02*)	0.05 (*0.03*)	0.15 (*0.02*)	0.10 (*0.03*)
Pa PD82.5*	9.23 (*0.30*)	7.11 (*0.53*)	7.64 (*0.55*)	7.28 (*0.61*)	7.18 (*0.80*)	7.03 (*0.55*)	7.50 (*0.29*)	6.07 (*0.57*)	5.95 (*0.47*)	6.01 (*0.34*)	6.08 (*0.48*)	6.61 (*0.33*)	1.55 (*0.33*)	0.17 (*0.06*)	0.44 (*0.09*)	0.30 (*0.24*)	0.54 (*0.06*)	0.35 (*0.08*)
Pa PD96.4	10.22 (*0.08*)	6.99 (*0.32*)	7.74 (*0.28*)	7.58 (*0.14*)	7.50 (*0.32*	7.53 (*0.26*)	6.95 (*0.24*)	6.53 (*0.33*)	6.33 (*0.39*)	6.70 (*0.26*)	6.51 (*0.35*)	6.83 (*0.30*)	0.97 (*0.12*)	0.27 (*0.02*)	0.20 (*0.03*)	0.14 (*0.03*)	0.2 (*0.07*)	0.11 (*0.02*)
Se PD6	8.43 (*0.51*)	2.76 (*1.79*)	5.73 (*0.29*)	3.73 (*1.12*)	5.31 (*0.28*)	6.31 (*0.11*)	7.47 (*0.51*)	4.02 (*0.5*)	3.52 (*0.62*)	5.63 (*0.12*)	5.14 (*0.13*)	6.17 (*0.15*)	0.55 (*0.23*)	0.07 (*0.04*)	0.05 (*0.02*)	0.02 (*0*)	0.05 (*0.03*)	0.05 (*0.03*)
Se PD10.2	8.66 (*0.15*)	4.23 (*0.15*)	6.87 (*0.24*)	7.05 (*0.14*)	6.83 (*0.22*)	7.07 (*0.21*)	8.10 (*0.25*)	4.64 (*0.15*)	5.91 (*0.19*)	5.27 (*0.12*)	6.15 (*0.4*)	6.45 (*0.14*)	1.21 (*0.28*)	0.05 (*0.01*)	0.06 (*0.03*)	0.02 (*0*)	0.02 (*0.01*)	0.04 (*0.01*)
Se	8.3	4.54	6.87	6.78	6.60	7.09	7.93	4.34	6.01	6.03	5.79	6.13	0.61	0.04	0.05	0.02	0.03	0.04

PD11.4	*(0.14)*	*(0.34)*	*(0.14)*	*(0.28)*	*(0.4)*	*(0.14)*	*(0.2)*	*(0.27)*	*(0.22)*	*(0.3)*	*(0.16)*	*(0.2)*	*(0.29)*	*(0.02)*	*(0.02)*	*(0)*	*(0.02)*	*(0.01)*
Se PD20.8	7.32 *(0.2)*	5.01 *(0.16)*	6.47 *(0.03)*	6.75 *(0.25)*	6.62 *(0.25)*	6.53 *(0.14)*	7.72 *(0.07)*	5.54 *(0.18)*	6.56 *(0.16)*	6.54 *(0.08)*	6.68 *(0.17)*	5.74 *(0.18)*	0.27 *(0.08)*	0.13 *(0.02)*	0.10 *(0.02)*	0.05 *(0.02)*	0.09 *(0.02)*	0.05 *(0.02)*
Se PD36.1*	8.21 *(0.32)*	4.23 *(0.41)*	6.23 *(0.37)*	6.5 *(0.31)*	6.07 *(0.24)*	6.76 *(0.17)*	7.88 *(0.32)*	3.90 *(0.41)*	6.18 *(0.31)*	6.44 *(0.17)*	5.75 *(0.24)*	5.91 *(0.37)*	0.38 *(0.15)*	0.06 *(0.02)*	0.05 *(0.03)*	0.02 *(0)*	0.04 *(0.02)*	0.04 *(0.03)*
Se PD45.8	8.48 *(0.52)*	4.8 *(0.82)*	5.82 *(0.56)*	6.34 *(0.35)*	6.21 *(0.23)*	6.5 *(0.34)*	7.93 *(0.34)*	4.58 *(0.11)*	6.52 *(0.18)*	6.31 *(0.14)*	6.54 *(0.16)*	6.56 *(0.13)*	0.8 *(0.39)*	0.08 *(0.02)*	0.06 *(0.06)*	0.03 *(0.02)*	0.12 *(0.05)*	0.08 *(0.02)*
Se PD62.7	7.99 *(0.17)*	5.14 *(0.23)*	5.82 *(0.66)*	6.32 *(0.2)*	6.76 *(0.42)*	6.18 *(0.22)*	7.84 *(0.25)*	4.91 *(0.27)*	6.22 *(0.27)*	5.47 *(0.16)*	6.31 *(0.41)*	6.66 *(0.15)*	0.80 *(0.27)*	0.11 *(0.04)*	0.05 *(0.03)*	0.04 *(0.03)*	0.06 *(0.02)*	0.14 *(0.09)*
Se PD64.6	8.33 *(0.16)*	4.93 *(0.21)*	5.71 *(0.28)*	6.71 *(0.18)*	6.32 *(0.21)*	6.99 *(0.08)*	7.88 *(0.16)*	4.57 *(0.24)*	6.39 *(0.24)*	6.17 *(0.26)*	6.32 *(0.29)*	6.5 *(0.20)*	0.6 *(0.11)*	0.07 *(0.02)*	0.12 *(0.05)*	0.02 *(0)*	0.13 *(0.08)*	0.06 *(0)*
Se PD85.8	8.27 *(0.13)*	3.18 *(0.63)*	6.46 *(0.21)*	6.46 *(0.23)*	6.44 *(0.13)*	6.74 *(0.12)*	7.75 *(0.21)*	5.4 *(0.13)*	6.39 *(0.11)*	5.61 *(0.52)*	5.99 *(0.21)*	6.23 *(0.14)*	0.75 *(0.2)*	0.05 *(0.03)*	0.08 *(0.02)*	0.02 *(0)*	0.07 *(0.04)*	0.07 *(0.02)*
Se PD97	8.26 *(0.12)*	5.6 *(0.19)*	6.92 *(0.20)*	7.2 *(0.17)*	6.71 *(0.21)*	7.03 *(0.09)*	8.09 *(0.17)*	5.84 *(0.21)*	6.25 *(0.30)*	6.99 *(0.18)*	6.39 *(0.14)*	7.02 *(0.06)*	0.15 *(0.09)*	0.08 *(0.02)*	0.03 *(0.01)*	0.05 *(0.03)*	0.08 *(0.02)*	0.17 *(0.04)*
Sha PD10.1	8.12 *(0.26)*	4.73 *(0.08)*	4.93 *(0.44)*	4.97 *(0.13)*	5.06 *(0.18)*	5.46 *(0.45)*	7.46 *(0.17)*	4.62 *(0.61)*	6.06 *(0.26)*	6.38 *(0.24)*	5.78 *(0.16)*	6.26 *(0.18)*	0.37 *(0.21)*	0.16 *(0.03)*	0.13 *(0.06)*	0.08 *(0.06)*	0.11 *(0.02)*	0.11 *(0.04)*
Sha PD20.7	7.47 *(0.20)*	3.7 *(0.26)*	3.30 *(0.58)*	5.16 *(0.57)*	5.40 *(0.52)*	5.68 *(0.48)*	7.28 *(0.25)*	4.61 *(0.44)*	5.71 *(0.14)*	6.18 *(0.16)*	5.82 *(0.17)*	6.27 *(0.17)*	0.13 *(0.06)*	0.13 *(0.06)*	0.08 *(0.05)*	0.11 *(0.06)*	0.16 *(0.12)*	0.2 *(0.06)*
Sha PD50.1	7.34 *(0.23)*	2.42 *(0.26)*	3.94 *(1.13)*	4.7 *(0.63)*	6.14 *(0.83)*	5.41 *(0.26)*	6.97 *(0.2)*	4.26 *(0.43)*	5.45 *(0.21)*	5.99 *(0.16)*	5.8 *(0.26)*	5.8 *(0.22)*	0.04 *(0.01)*	0.02 *(0)*	0.02 *(0.01)*	0.01 *(0)*	0.02 *(0.01)*	0.02 *(0.01)*
Sho PD37.7	7.53 *(0.29)*	3.97 *(0.81)*	3.75 *(0.8)*	5.04 *(0.16)*	4.98 *(0.4)*	5.25 *(0.27)*	6.71 *(0.53)*	4.92 *(0.26)*	5.33 *(0.24)*	5.77 *(0.17)*	5.65 *(0.25)*	5.81 *(0.21)*	0.03 *(0.03)*	0.02 *(0.01)*	0.02 *(0.01)*	0.01 *(0.01)*	0.06 *(0.05)*	0.04 *(0.03)*
Scc PD42.5	8.46 *(0.13)*	3.44 *(0.22)*	5.53 *(0.2)*	6.47 *(0.17)*	6.5 *(0.24)*	6.46 *(0.15)*	7.89 *(0.14)*	3.77 *(0.28)*	5.68 *(0.18)*	5.66 *(0.18)*	5.56 *(0.18)*	5.49 *(0.30)*	0.48 *(0.16)*	0.05 *(0.02)*	0.03 *(0.02)*	0.02 *(0)*	0.05 *(0.02)*	0.05 *(0.03)*
Scc PD81.1	8.17 *(0.34)*	4.89 *(0.46)*	6.69 *(0.12)*	6.90 *(0.30)*	6.86 *(0.41)*	6.64 *(0.37)*	7.42 *(0.23)*	4.71 *(0.98)*	6.24 *(0.15)*	6 *(0.43)*	6.19 *(0.30)*	6.27 *(0.37)*	0.34 *(0.11)*	0.07 *(0.01)*	0.11 *(0.04)*	0.03 *(0.02)*	0.07 *(0.03)*	0.06 *(0.03)*

All the strains are from different catheters. *, Strains derived from catheters of patients with infection. *Pa*, *P. aeruginosa*; *Se*, *S. epidermidis*, *Scc, S. caprae/capitis*; *Sha*, *S. haemolyticus*; *Sho*, *S. hominis*; C+, positive control, TSB culture medium, C-, negative control, saline. Values are mean with standard deviation in italic within parenthesis.
